# The Heart-Brain Connection: Acute Intracranial Pressure Elevation as a Cause of Isolated Bradycardia

**DOI:** 10.7759/cureus.105239

**Published:** 2026-03-14

**Authors:** Aamer Naofal, Kamran Zaheer, Simon Pinsky, Stephen Keim, Aditya Saini

**Affiliations:** 1 Cardiology, University of Florida College of Medicine-Jacksonville, Jacksonville, USA; 2 Electrophysiology, University of Florida College of Medicine-Jacksonville, Jacksonville, USA

**Keywords:** cushing reflex, elevated intracranial pressure, intermittent sinus pause, lumbar puncture, reversible bradycardia

## Abstract

Bradyarrhythmias are commonly attributed to intrinsic conduction disease, ischemia, or medication effects; however, not all etiologies are cardiac in origin. Acute elevation in intracranial pressure (ICP) can provoke clinically significant bradycardia, sometimes without the complete Cushing triad. We describe a 50-year-old nonverbal man with marginal zone lymphoma on rituximab, prior cerebrovascular accident, seizure disorder, and chronic hydrocephalus managed with a ventriculoperitoneal (VP) shunt who presented with lethargy and hypotension during rituximab infusion and was found to have pneumonia with acute hypoxemic respiratory failure (AHRF). During hospitalization, he developed recurrent profound bradycardia with sinus pauses on telemetry. Standard reversible cardiac causes were addressed and ruled out; however, given his history of hydrocephalus and concern for intermittent ICP elevation, a lumbar puncture was performed, which demonstrated an opening pressure of 22 cm H₂O. Therapeutic drainage of 28 cc of cerebrospinal fluid (CSF) resulted in immediate and durable resolution of bradycardia with stable telemetry thereafter. This case highlights acute ICP elevation as a reversible and underrecognized cause of clinically significant bradyarrhythmia and underscores the importance of considering central drivers of bradycardia in patients with neurologic comorbidity to avoid unnecessary permanent device implantation.

## Introduction

Bradycardia in hospitalized patients typically prompts evaluation for myocardial ischemia, medication effects, metabolic abnormalities, or intrinsic conduction system disease [1]. When recurrent or severe, electrophysiology consultation and consideration of pacing therapy are often pursued. Less commonly, bradyarrhythmias arise from neurogenic mechanisms related to elevated intracranial pressure (ICP), mediated through brainstem autonomic pathways that influence cardiac rhythm [2,3]. Elevations in ICP can alter autonomic output through brainstem centers such as the nucleus tractus solitarius and dorsal vagal nucleus, resulting in increased parasympathetic tone and suppression of sinoatrial node activity. Although classically described as part of the Cushing response, isolated bradycardia may occur even in the absence of accompanying hypertension or respiratory changes [3,4]. Recognition of this mechanism is clinically important, as treatment of the underlying intracranial process may reverse the arrhythmia and obviate invasive cardiac interventions [5].

## Case presentation

A 50-year-old nonverbal man with a history of marginal zone lymphoma on rituximab therapy, prior cerebrovascular accident with residual neurologic deficits, seizure disorder, chronic failure to thrive, and chronic hydrocephalus managed with a ventriculoperitoneal (VP) shunt presented to the emergency department after developing lethargy and hypotension during a scheduled rituximab infusion. He had tolerated prior infusions without complication, and there was concern that his symptoms preceded the infusion rather than representing an acute infusion reaction.

On presentation, he was hypoxic and required endotracheal intubation for acute hypoxemic respiratory failure (AHRF). He was treated for sepsis with broad-spectrum antibiotics and supportive care, with subsequent improvement in respiratory status allowing for extubation. After initial resuscitation and treatment of sepsis, the patient continued to experience intermittent hypotension requiring vasopressor support, including during periods of normal sinus rhythm.

**Figure 1:**
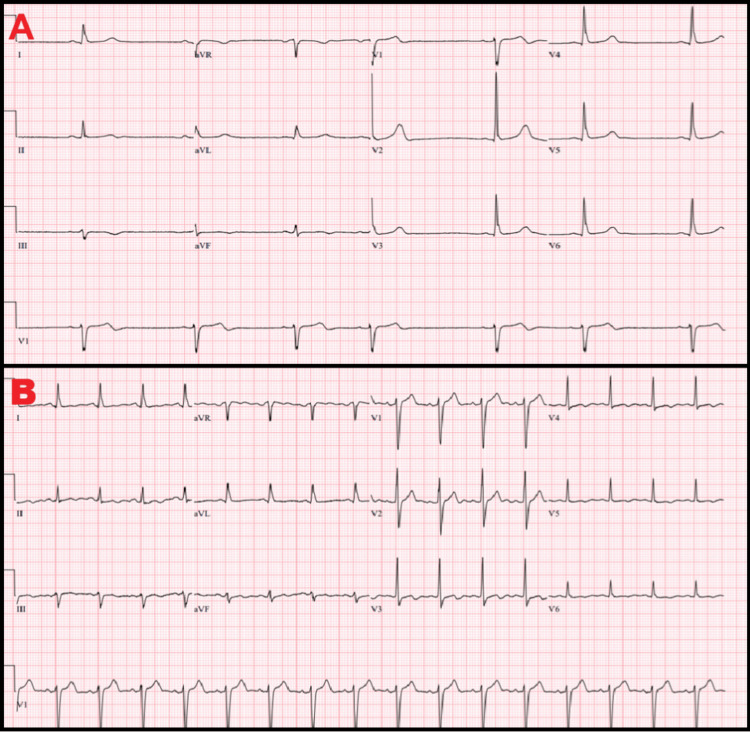
(A) Prelumbar puncture ECG demonstrating sinus bradycardia with preserved atrioventricular conduction. (B) Postlumbar puncture ECG showing restoration of normal sinus rhythm with resolution of bradycardia

During the subsequent hospital course, he developed recurrent episodes of marked bradycardia noted on continuous telemetry monitoring. These episodes were characterized by sinus bradycardia with heart rates frequently in the 30-40 bpm range, and intermittent sinus pauses lasting up to several seconds. Events occurred most frequently during periods of rest and sleep, but were also observed during daytime hours. When not bradycardic, the patient was in normal sinus rhythm with intact atrioventricular conduction. Continuous telemetry did not demonstrate high-grade atrioventricular block or malignant ventricular arrhythmias. Representative prelumbar puncture electrocardiography demonstrating significant sinus bradycardia with preserved atrioventricular conduction and sinus arrhythmia is shown in Figure 1A.

Cardiac evaluation demonstrated preserved cardiac function without structural abnormalities to explain the arrhythmia. Atrioventricular nodal blocking agents were not administered, electrolyte abnormalities, including potassium and magnesium, were corrected, and other reversible metabolic causes of bradyarrhythmia were excluded. Notably, hypotension persisted during periods of normal sinus rhythm and did not consistently coincide with bradycardic episodes, suggesting that the rhythm disturbance was not the primary driver of the patient’s hemodynamic instability.

Given the patient’s history of chronic hydrocephalus and VP shunt dependence, intermittent elevation in intracranial pressure (ICP) was suspected as a contributor to the recurrent bradycardia. Lumbar puncture demonstrated an opening pressure of 22 cm H₂O. Therapeutic drainage of 28 cc of cerebrospinal fluid (CSF) was performed, resulting in immediate resolution of bradycardia and sinus pauses. Telemetry remained stable for the remainder of hospitalization, and no pacing therapy was required. Postlumbar puncture electrocardiography demonstrated restoration of normal sinus rhythm (Figure 1B).

## Discussion

In this patient, the recurrence of sinus pauses and their immediate resolution following CSF drainage suggest a direct relationship between ICP modulation and cardiac rhythm [2]. The temporal association observed after lumbar puncture supports a centrally mediated and reversible etiology.

Intermittent ICP elevations in individuals with chronic hydrocephalus or CSF diversion devices may be clinically subtle [6]. Fluctuations in central autonomic tone, mediated through brainstem and higher regulatory pathways, can manifest as sinus bradycardia or pauses [7,8]. In the absence of clear neurologic deterioration, rhythm disturbance may serve as an early physiologic marker of altered intracranial dynamics.

This case underscores the importance of maintaining diagnostic breadth in patients with neurologic comorbidity who develop unexplained bradyarrhythmia. In select circumstances, evaluation of ICP may be warranted before pursuing invasive cardiac intervention [1].

## Conclusions

Acute elevation of ICP may present as isolated, clinically significant bradycardia with sinus pauses in the absence of classic neurologic findings. In patients with hydrocephalus or VP shunts who develop unexplained recurrent bradyarrhythmias, central nervous system causes should be considered alongside cardiac etiologies. Targeted CSF drainage may promptly restore normal cardiac rhythm and prevent unnecessary permanent device implantation.
